# Supplementary material for: Cyberattacks in supply chains: A multi-case study
Source: PLoS One. 2026 May 22;21(5):e0350010. doi: 10.1371/journal.pone.0350010 (PMC13196919; doi:10.1371/journal.pone.0350010)
Supplement: S1 File — (DOCX) [file pone.0350010.s001.docx]

# Supporting information

**S1 Table. Summary of data sources**

| **Case** | **Data Source Category** | **Key Document / Author** | **URL** |
| --- | --- | --- | --- |
| **A: Target** | Academic Case Study | Plachkinova and Maurer (2018) | <http://jise.org/Volume29/n1/JISEv29n1p11.html> |
|  | Government Report | U.S. Senate Commerce Committee | [commerce.senate.gov/services/files/24d3c229-4f2f-405d-b8db-a3a67f183883](https://www.commerce.senate.gov/services/files/24d3c229-4f2f-405d-b8db-a3a67f183883) |
|  | Corporate Statement | Target Corp Press Release | [corporate.target.com/press/release/2013/12/target-confirms-unauthorized-access-to-payment-card-data-in-u-s-stores](https://corporate.target.com/press/release/2013/12/target-confirms-unauthorized-access-to-payment-card-data-in-u-s-stores) |
| **B: Maersk** | Investigative Journalism | Andy Greenberg (Wired) | [wired.com/story/notpetya-cyberattack-ukraine-russia-code-crashed-the-world/](https://www.wired.com/story/notpetya-cyberattack-ukraine-russia-code-crashed-the-world/) |
|  | Technical Analysis | Microsoft Security Blog | [microsoft.com/en-us/security/blog/2017/10/03/advanced-threat-analytics-security-research-network-technical-analysis-notpetya/](https://www.microsoft.com/en-us/security/blog/2017/10/03/advanced-threat-analytics-security-research-network-technical-analysis-notpetya/) |
|  | Corporate Report | Maersk Investor Update | [investor.maersk.com/news-releases/news-release-details/cyber-attack-update](https://investor.maersk.com/news-releases/news-release-details/cyber-attack-update) |
| **C: SolarWinds** | Government Directive | CISA Emergency Directive 21-01 | [cisa.gov/news-events/directives/ed-21-01-mitigate-solarwinds-orion-code-compromise](https://www.google.com/search?q=https://www.cisa.gov/news-events/directives/ed-21-01-mitigate-solarwinds-orion-code-compromise) |
|  | Technical Forensic | FireEye/Google Mandiant | [cloud.google.com/blog/topics/threat-intelligence/sunburst-additional-technical-details/](https://cloud.google.com/blog/topics/threat-intelligence/sunburst-additional-technical-details/) |
| **D: Colonial** | Government Directive | TSA Cybersecurity Directive | [fox5ny.com/news/tsa-updates-cybersecurity-directive-after-colonial-pipeline-ransomware-attack](https://www.google.com/search?q=https://www.fox5ny.com/news/tsa-updates-cybersecurity-directive-after-colonial-pipeline-ransomware-attack) |
|  | Investigative Journalism | Reuters (Wolf) | [thomsonreuters.com/en-us/posts/investigation-fraud-and-risk/colonial-pipeline-ransom-funds](https://www.thomsonreuters.com/en-us/posts/investigation-fraud-and-risk/colonial-pipeline-ransom-funds) |
| **E: Quanta** | Investigative Journalism | Wired (Newman) | [wired.com/story/apple-ransomware-attack-quanta-computer](https://www.wired.com/story/apple-ransomware-attack-quanta-computer) |
|  | Threat Intelligence | BleepingComputer (Gatlan) | [bleepingcomputer.com/news/security/revil-gang-tries-to-extort-apple-threatens-to-sell-stolen-blueprints/](https://www.bleepingcomputer.com/news/security/revil-gang-tries-to-extort-apple-threatens-to-sell-stolen-blueprints/) |
| **F: MediBank** | Government/Legal | OAIC Concise Statement | [oaic.gov.au/__data/assets/pdf_file/0025/221974/Australian-Information-Commissioner-v-Medibank-Private-Limited-concise-statement.pdf](https://www.oaic.gov.au/__data/assets/pdf_file/0025/221974/Australian-Information-Commissioner-v-Medibank-Private-Limited-concise-statement.pdf) |
|  | Investigative Journalism | ABC News Australia | [abc.net.au/news/2024-06-17/biz-medibank-court-absence-of-multi-factor-authentication/103987732](https://www.abc.net.au/news/2024-06-17/biz-medibank-court-absence-of-multi-factor-authentication/103987732) |
| **G: Toyota** | Investigative Journalism | New York Times (2022) | [nytimes.com/2022/02/28/business/toyota-stoppage-cyberattack.html](https://www.nytimes.com/2022/02/28/business/toyota-stoppage-cyberattack.html) |
|  | Journalism/Industry | InfoSecurity Magazine | [infosecurity-magazine.com/news/toyota-production-japan-ransomware](https://www.infosecurity-magazine.com/news/toyota-production-japan-ransomware) |

**S2. List of Candidate Supply Chain Cyberattack Cases**

The following list presents the 49 candidate supply chain cyberattack cases (2010–2025) identified for the candidate pool. These cases were selected to represent diverse sectors and attack mechanisms, providing the theoretical basis for the study's replication logic. To ensure academic and forensic rigor, all URLs are restricted to government advisories, regulatory filings, official corporate reports, and reputable news organizations.

1. Stuxnet (2010): https://docs.broadcom.com/docs/security-response-w32-stuxnet-dossier-11-en
2. Operation Aurora (2010): https://www.sec.gov/Archives/edgar/data/1288776/000119312510005667/dex991.htm
3. Target Corporation Data Breach (2013): https://www.commerce.senate.gov/services/files/24d3c229-4f2f-405d-b8db-a3a67f183883
4. Dragonfly / Energetic Bear (2014): https://attack.mitre.org/groups/G0035/
5. Anthem Health Insurance (2015): https://www.justice.gov/opa/pr/four-members-chinese-military-hacking-group-indicted-hacking-anthem-and-three-other-us
6. Adobe Systems Source Code Theft (2013): https://www.csoonline.com/article/553655/adobe-breach-the-knowns-and-unknowns.html
7. Apple XCodeGhost (2015): https://www.paloaltonetworks.com/apps/pan/public/downloadResource?pagePath=/content/pan/en_US/resources/white-papers-and-guides/xcodeghost-malware-analysis
8. Premera Blue Cross (2015): https://www.reuters.com/article/us-premera-cyberattack-idUSKBN0ME29E20150317
9. Yahoo Data Breach (2013–2016): https://www.justice.gov/opa/pr/two-russian-fsb-officers-and-two-criminal-hackers-indicted-2014-hack-yahoo-networks
10. Operation Socialist / Belgacom (2010–2013): https://www.spiegel.de/international/europe/british-spy-agency-gchq-hacked-belgian-telecoms-firm-a-923406.html
11. Subway POS Hacking Scheme (2012): https://www.justice.gov/archives/opa/pr/former-subway-franchise-owner-sentenced-18-months-prison-gift-card-hacking-scheme-subway
12. Global Payments / Visa-Mastercard Processor Breach (2012): https://www.sec.gov/Archives/edgar/data/1123360/000112336012000014/gpn-20120330xex991.htm
13. Goodwill Industries Third-Party Vendor Breach (2014): https://www.securityweek.com/goodwill-blames-credit-card-breach-third-party-vendor/
14. Maersk / NotPetya (2017): https://investor.maersk.com/news-releases/news-release-details/cyber-attack-update
15. Equifax Data Breach (2017): https://www.gao.gov/assets/gao-18-559.pdf
16. CCleaner / Kingslayer (2017): https://www.darkreading.com/cyberattacks-data-breaches/ccleaner-compromised-to-distribute-malware
17. WannaCry Ransomware (2017): https://www.cisa.gov/news-events/alerts/2017/05/12/wannacry-ransomware
18. TSMC Manufacturing Software Infection (2018): https://www.reuters.com/article/us-tsmc-virus-idUSKBN1KQ0B5
19. ASUS ShadowHammer (2019): https://www.wired.com/story/asus-software-update-hack/
20. SolarWinds Orion Sunburst (2020): https://www.cisa.gov/news-events/directives/ed-21-01-mitigate-solarwinds-orion-code-compromise
21. VeraPort Download Verification Compromise (2020): https://www.dni.gov/files/NCSC/documents/supplychain/Software_Supply_Chain_Attacks.pdf
22. Twilio SDK Code Injection (2020): https://www.zdnet.com/article/twilio-says-hackers-modified-one-of-its-javascript-libraries/
23. GoldenSpy Embedded Backdoor (2020): https://attack.mitre.org/software/S0493/
24. Wipro IT Services Phishing Platform (2020): https://www.reuters.com/article/us-wipro-cyber/wipro-says-its-systems-breached-by-advanced-phishing-attack-idUSKCN1RS1K8/
25. Codecov Docker Image Compromise (2020): https://www.reuters.com/technology/exclusive-investigators-find-hackers-breached-codecov-software-months-ago-2021-04-15/
26. Kaseya VSA Ransomware Propagation (2021): https://www.cisa.gov/news-events/alerts/2021/07/04/kaseya-vsa-ransomware-incident
27. Colonial Pipeline Energy Infrastructure (2021): https://www.tsa.gov/newsroom/tsa-announces-new-cybersecurity-requirements-critical-pipeline-owners-and-operators
28. Quanta Computer Dual Extortion (2021): https://www.bloomberg.com/news/articles/2021-04-20/revil-ransomware-gang-demands-50-million-from-apple-supplier
29. MediBank Third-Party Credential Theft (2022): https://www.oaic.gov.au/news-and-events/news-and-media-releases/commissioner-commences-court-action-against-medibank
30. Toyota / Kojima Industries JIT Halt (2022): https://www.nytimes.com/2022/02/28/business/toyota-stoppage-cyberattack.html
31. MOVEit Transfer Zero-Day Exploitation (2023): https://www.cisa.gov/news-events/cybersecurity-advisories/aa23-158a
32. Okta Support System Credential Breach (2023): https://www.reuters.com/technology/okta-says-hackers-stole-information-all-customer-support-users-2023-11-29/
33. 3CX Desktop App Cascading Compromise (2023): https://www.reuters.com/technology/hackers-behind-3cx-breach-used-cascading-supply-chain-attack-2023-04-20/
34. Mimecast Authentication Certificate Breach (2021): https://www.sec.gov/files/litigation/admin/2024/33-11322.pdf
35. JetBrains TeamCity Build Pipeline Vulnerability (2023): https://www.cisa.gov/news-events/cybersecurity-advisories/aa23-347a
36. Applied Materials Semiconductor Supply Chain (2023): https://www.reuters.com/technology/applied-materials-warns-of-250-mln-loss-from-supply-chain-cyber-attack-2023-02-16/
37. Airbus Vendor Data Disclosure (2023): https://www.securityweek.com/airbus-confirms-cyberattack-after-hacker-posts-vendor-data/
38. Salesloft-Drift / Salesforce OAuth Poisoning (2025): https://www.infosecurity-magazine.com/news-features/top-10-cyberattacks-of-2025/
39. Miljödata HR SaaS Ransomware (2025): https://blackcell.io/data-protection-incidents-related-to-the-supply-chain-in-2025/
40. Jaguar Land Rover Production Disruption (2025): https://www.reuters.com/business/autos-transportation/jaguar-land-rover-production-hit-by-cyber-attack-2025-09-01/
41. Asahi Breweries Operational Technology Halt (2025): https://www.reuters.com/business/retail-consumer/asahi-breweries-halts-production-after-cyberattack-2025-09-28/
42. Marks & Spencer Third-Party Contractor Breach (2025): https://www.infosecurity-magazine.com/news/marks-spencer-hit-by-targeted-cyberattack/
43. National Defense Corporation (NDC) Logistics Leak (2025): https://www.csoonline.com/article/3542289/national-defense-corp-hacked-by-interlock-group.html
44. Shai-Hulud npm Self-Propagating Worm (2025): https://owasp.org/Top10/2025/A03_2025-Software_Supply_Chain_Failures/
45. Trust Wallet Chrome Extension Supply Chain Attack (2025): https://www.csis.org/programs/strategic-technologies-program/significant-cyber-incidents
46. Coupang E-commerce Administrative Access Breach (2025): https://www.csis.org/programs/strategic-technologies-program/significant-cyber-incidents
47. Ingram Micro Global IT Distribution Outage (2025): https://ir.ingrammicro.com/press-releases/detail/945/ingram-micro-issues-statement-regarding-cybersecurity-incident
48. XZ Utils liblzma Backdoor (2024): https://www.cisa.gov/news-events/alerts/2024/03/29/reported-vulnerability-liblzma-affecting-xz-utils-cve-2024-3094
49. Yanfeng Cyberattack Impacts Stellantis (2023): https://thecyberexpress.com/cyberattack-on-yanfeng-impacts-jeep-dodge-ram/
