# Supplementary material for: Cyberattacks in supply chains: A multi-case study
Source: PLoS One. 2026 May 22;21(5):e0350010. doi: 10.1371/journal.pone.0350010 (PMC13196919; doi:10.1371/journal.pone.0350010)
Supplement: Attachment — Submitted filename: Response to R1 reivews.pdf [file pone.0350010.s002.pdf]

Response to Editor Comments

We thank you and the four referees for the detailed reviews that have helped us greatly in revising the manuscript. Following your suggestions, we have made significant changes to our manuscript. Together with the new version of the manuscript, we are also submitting revision notes for the review team. In our revision, we have made conscientious efforts to address your concerns. We believe that by addressing the review team's concerns, we now have a much stronger manuscript. In the following paragraphs, the comments that you raise are reproduced verbatim in *italics*. Our responses to the comments are in regular font. We have also submitted a revised manuscript with tracked changes.

1. Please ensure that your manuscript meets PLOS ONE's style requirements, including those for file naming. The PLOS ONE style templates can be found at https://journals.plos.org/plosone/s/file?id=wjVg/PLOSOne_formatting_sample_main_body.pdf and https://journals.plos.org/plosone/s/file?id=ba62/PLOSOne_formatting_sample_title_authors_affiliations.pdf

- Thank you for the instructions. We revised the manuscript to comply with PLOS ONE formatting and file-naming requirements.

2. Thank you for stating the following in the Acknowledgments Section of your manuscript: "The authors received no specific funding for this work." We note that you have provided funding information that is not currently declared in your Funding Statement. However, funding information should not appear in the Acknowledgments section or other areas of your manuscript. We will only publish funding information present in the Funding Statement section of the online submission form.

Please remove any funding-related text from the manuscript and let us know how you would like to update your Funding Statement. Currently, your Funding Statement reads as follows: "The author(s) received no specific funding for this work." Please include your amended statements within your cover letter; we will change the online submission form on your behalf.

- Per your comment, we removed the funding-related text from the manuscript and confirm that no funding information needs to be added to the Funding Statement.

3. Please note that your Data Availability Statement is currently missing the repository name and/or the DOI/accession number of each dataset OR a direct link to access each database. If your manuscript is accepted for publication, you will be asked to provide these details on a very short timeline. We therefore suggest that you provide this information now, though we will not hold up the peer review process if you are unable.

- We updated the Data Availability Statement to include the repository name and DOI.

4. We note you have included a table to which you do not refer in the text of your manuscript. Please ensure that you refer to Table 3 in your text; if accepted, production will need this reference to link

the reader to the Table.

- Thank you for pointing this out. We corrected the in-text table references in Section 5 so that Tables 2 and 3 are now cited appropriately.

5. If the reviewer comments include a recommendation to cite specific previously published works, please review and evaluate these publications to determine whether they are relevant and should be cited. There is no requirement to cite these works unless the editor has indicated otherwise.

- Thank you for your guidance. There is no such recommendation in the reviewer comments.

Additional Editor Comments:

The manuscript investigates the dynamics of Supply Chain Cyberattacks (SCCAs) through a qualitative multi-case study of seven major cyber incidents. The authors apply the SCCSS framework, which conceptualizes cybersecurity across three interconnected subsystems:
- IT security
- organizational security
- supply chain security.
Using secondary data sources such as government reports, forensic analyses, corporate disclosures, and investigative journalism, the study maps attack vectors and propagation mechanisms across these subsystems. The findings suggest that:
- most cyberattacks originate from third-party connections within supply chains,
- attack propagation is often amplified by coordination failures across systems,
- resilience improves when organizations implement information sharing, internal controls, and coordinated governance mechanisms.
The paper concludes that effective cybersecurity governance requires a system-level perspective rather than isolated defensive measures.

- Thank you for the comprehensive summary of our manuscript.

The topic is highly relevant given the increasing importance of supply chain cybersecurity. The manuscript offers a useful synthesis of several prominent incidents. However, the current version presents important limitations regarding theoretical positioning, methodological transparency, and analytical depth.

- Thank you for acknowledging the relevance of our topic. We revised Sections 1 and 6.1 to clarify the study's theoretical contribution and to specify how the revised analysis extends the SCCSS framework. Specifically, we have updated Section 5.9 to verify the propositions and discuss how they explain the variations among cases.

The manuscript claims to extend the SCCSS framework and contribute to the understanding of supply chain cyber risk. However, the theoretical contribution remains unclear. For instance, the introduction states that the study aims to examine: "how system coordination and information sharing enhance resilience by establishing a system-level defense."

However, the paper mainly applies the SCCSS framework rather than substantially extending it. The analysis largely maps observed events to existing categories (IT, organizational, supply chain security).
Consequently, the manuscript would benefit from clarifying:
- what specific theoretical advancement is achieved,
- whether the study tests, refines, or extends the SCCSS framework,
- how the findings advance existing literature on cyber resilience or supply chain risk governance.
Without this clarification, the contribution risks appearing primarily descriptive.

- We appreciate the reviewer's critical assessment regarding the theoretical depth of the study. We have revised the manuscript (Section 6.1 and Section 1) to clarify that this research moves beyond a descriptive application of the SCCSS framework to offer a structural refinement and extension of the model.

- <u>Specific Theoretical Advancement: The Synergy Dependency</u>: Our analysis suggests a synergy dependency among the SCCSS subsystems, in which external relational governance is more effective when supported by internal organizational readiness. We revised the manuscript to present this as a theoretical refinement rather than a categorical claim.

- <u>Extension of the SCCSS Framework: PoPs as Transmission Sites</u>. We also revised the SCCSS framework discussion to treat the Points of Penetration as transmission sites that help explain how cyber incidents propagate across subsystems. While the original SCCSS model identifies these as connection points, our manuscript redefines them as dynamic mechanisms that dictate the velocity and impact region of an attack. This extension allows the framework to account for how digital failures at a peripheral node convert into systemic physical halts, as evidenced by the Toyota and Maersk incidents.

- <u>Advancing Literature on Cyber Resilience and Governance.</u> The findings advance existing literature by bridging the gap between digital and physical risk management within the Supply Chain Risk Management (SCRM) domain. We demonstrate that physical "Lean" optimizations, such as Just-in-Time models, create rigid digital dependencies that accelerate the spread of disruption—a phenomenon we define as Operational Coupling. By integrating Supply Chain Governance (SCG) theory with the SCCSS framework, this study provides a verified theoretical basis for future research into digital resilience as a systemic property emerging from the interdependence of infrastructure, culture, and external relationships.

The methodology section describes a qualitative multi-case study based on seven well-known cyber incidents. While the authors mention that cases were selected to represent diverse sectors and attack types, the selection criteria remain insufficiently justified.
For example, the paper states: "Selection criteria required that each case involve a cyberattack targeting these subsystems, offer rich verifiable data, and reflect diverse sectors and motives."
However, the manuscript does not explain:
- how many candidate cases were initially considered,
- whether any systematic sampling strategy was used,
- whether the cases represent theoretical replication or convenience sampling.

Since the study focuses on high-profile incidents, there is a risk of selection bias, which should be explicitly discussed.

- We agree with your comment and have added clarification in Section 3.2, page 10. Our candidate incidents are outlined in S2 List in Supporting Information.

The empirical analysis relies entirely on publicly available secondary sources, including:
- investigative journalism
- corporate reports
- government documents
- forensic analyses.
While triangulation is mentioned, the manuscript does not fully discuss the limitations associated with this data source.
For example:
- journalistic reports may contain interpretive bias,
- technical reports may focus primarily on technical vulnerabilities rather than governance issues,
- corporate disclosures may omit sensitive details.
Although the authors acknowledge this limitation in the discussion, a deeper methodological reflection is necessary.

- We sincerely appreciate your critical assessment of our data sources. We agree that simply mentioning data triangulation is insufficient without critically reflecting on the inherent biases of the specific secondary sources utilized in our study.
- As you correctly pointed out, we have acknowledged this limitation in Section 6.3 (Limitations and Future Research Directions). The revised manuscript now explicitly details the specific limitations associated with our data types, acknowledging that journalistic accounts may harbor interpretive bias, forensic analyses often narrow their focus to technical flaws at the expense of governance context, and corporate disclosures are inherently designed to protect reputational assets and may omit sensitive operational details. We expanded Section 6.3 to discuss the limitations of journalistic, forensic, and corporate sources and to clarify the scope of the findings.

The manuscript presents detailed narrative descriptions of several incidents. For instance, the Quanta Computer case illustrates how attackers exploited supply chain relationships to access sensitive intellectual property. Similarly, the MediBank case describes credential theft and large-scale data exfiltration resulting from weak access controls. While these descriptions are informative, the analysis often remains descriptive rather than analytical. In many sections the paper primarily recounts the events and then maps them to SCCSS categories. Stronger analytical insights could be generated by:
- systematically comparing cases,
- identifying patterns across incidents,
- explaining why some attacks propagate more severely than others.

- We appreciate the reviewer's constructive feedback regarding the need for a more structured cross-case synthesis. To address this, we revised the Results section to include cross-case

comparison tables and a more explicit discussion of recurring propagation mechanisms. Specifically, we have added Table 4, which operationalizes key governance and resilience constructs across all seven incidents, mapping entry vectors, propagation mechanisms, and specific coordination failures. Furthermore, we have expanded Section 5.8 to explicitly define four primary propagation mechanisms (Network Flattening, Alert Paralysis, Operational Coupling, and Relational Weaponization), which provides the systematic pattern identification requested.

The manuscript states that cross-case synthesis was conducted to extract higher-level insights. However, the results section does not clearly present:
- structured cross-case comparisons,
- tables summarizing key variables,
- systematic pattern identification.
A comparative table summarizing the seven cases (e.g., entry vector, propagation mechanism, governance failure, mitigation response) would significantly improve clarity.

- We appreciate your helpful comment. We have added Table 4 and Table 5 to allow structured cross-case comparison and systematic pattern identification.

While the manuscript refers to several conceptual perspectives (e.g., supply chain governance theory, resilience and robustness concepts), the theoretical grounding of the study remains relatively limited. The analysis is primarily structured around the SCCSS framework, which functions more as an analytical classification model than as a fully developed theoretical lens. As a result, the manuscript would benefit from a stronger integration of established theories from supply chain management, organizational governance, or systems theory to better explain the mechanisms underlying cyberattack propagation and resilience. Furthermore, the SCCSS framework looks to be used primarily as coding tool and not as a theory building tool.

- We have added Supply Chain Governance theory to explain the underlying mechanisms. We have accordingly updated Section 4.2 and Section 5.9 to reflect this change in theoretical framework, which allows us to deepen the contrast and synthesis.

The manuscript does not clearly articulate explicit research questions. Explicitly stating the research questions would improve the structure of the study.

- Per your comment and Reviewer 4’s comment, we have revised the research questions. Please see our response to Reviewer 4’s first comment.

1. Is the manuscript technically sound, and do the data support the conclusions?

The manuscript must describe a technically sound piece of scientific research with data that supports the conclusions. Experiments must have been conducted rigorously, with appropriate controls, replication, and sample sizes. The conclusions must be drawn appropriately based on the data presented.

Reviewer #1: Yes

Reviewer #2: Partly

Reviewer #3: Partly

Reviewer #4: Partly

---

2. Has the statistical analysis been performed appropriately and rigorously?

Reviewer #1: Yes

Reviewer #2: Yes

Reviewer #3: Yes

Reviewer #4: N/A

---

3. Have the authors made all data underlying the findings in their manuscript fully available?

The PLOS Data policy requires authors to make all data underlying the findings described in their manuscript fully available without restriction, with rare exception (please refer to the Data Availability Statement in the manuscript PDF file). The data should be provided as part of the manuscript or its supporting information, or deposited to a public repository. For example, in addition to summary statistics, the data points behind means, medians and variance measures should be available. If there are restrictions on publicly sharing data—e.g. participant privacy or use of data from a third party—those must be specified.

Reviewer #1: Yes

Reviewer #2: Yes

Reviewer #3: Yes

Reviewer #4: Yes

---

4. Is the manuscript presented in an intelligible fashion and written in standard English?

PLOS ONE does not copyedit accepted manuscripts, so the language in submitted articles must be clear, correct, and unambiguous. Any typographical or grammatical errors should be corrected at revision, so please note any specific errors here.

Reviewer #1: Yes

Reviewer #2: Yes

Reviewer #3: No

Reviewer #4: Yes

5. Review Comments to the Author

Response to Reviewer #1 Comments

We thank you and the review team for the detailed reviews that have helped us greatly in revising the manuscript. Following your suggestions, we have made significant changes to our manuscript. Together with the new version of the manuscript, we are also submitting revision notes for the review team. In our revision, we have made conscientious efforts to address your concerns. We believe that by addressing the review team's concerns, we now have a much stronger manuscript. In the following paragraphs, the comments that you raise are reproduced verbatim in *italics*. Our responses to the comments are in regular font. We have also submitted a revised manuscript with tracked changes.

Although the manuscript addresses an important topic (supply chain cyberattacks), the conceptual novelty is limited. The study primarily applies the existing SCCSS framework to well-known public cyber incidents, without developing new theoretical constructs or analytical mechanisms.

- Thank you for the comment. Our contribution lies in refining the SCCSS framework through the concepts of synergy dependency, transmission sites, and propagation mechanisms. We identify a synergy dependency between subsystems and establish that the utility of external relational governance is functionally secondary to, and dependent upon, internal organizational readiness. Furthermore, by focusing on the points of penetration as the primary sites of cyber-physical conversion, the manuscript provides a new analytical mechanism to explain the transition from digital vulnerability to systemic physical paralysis. Finally, the introduction of four distinct propagation mechanisms offers the specific theoretical constructs needed to bridge the gap between isolated IT security and integrated supply chain resilience.

All case materials are derived from secondary public sources (media reports, government documents, and technical blogs). No primary data (e.g., interviews, internal reports, or practitioner surveys) are used, which weakens empirical depth and originality.

- We sincerely appreciate your critical assessment of our data sources. In our original manuscript, we acknowledged this limitation in Section 6.3 (Limitations and Future Research Directions). To address your comment, the revised manuscript now explicitly details the specific limitations associated with our data types, providing transparent and rigorous boundaries for our findings.

Case selection criteria require further clarification. The manuscript focuses exclusively on high-profile incidents (e.g., Target, SolarWinds, Maersk), which may introduce selection bias and limit generalizability to typical supply chain organizations.

- We agree with your comment and have added clarification in Section 3.2, page 11.

The SCCSS framework is adopted rather than substantially extended. The propositions (P1–P4) largely restate established concepts such as governance maturity, internal control, and information sharing, providing limited theoretical advancement.

- We have added Propositions 5 and 6. Moreover, we have incorporated Supply Chain Governance theory, which we believe improves the contributions of our propositions.

The causal relationships between governance mechanisms and cyber resilience remain largely inferential. The analysis does not rigorously distinguish correlation from causation across the multi-case comparisons.

- We agree with you on the causation versus correlation front, which is why we explicitly aimed for analytical findings rather than statistical generalization. Using replication logic across seven cases, we identified recurring patterns linking governance failures to similar propagation outcomes. Because the evidence is qualitative and based on secondary sources, we present these relationships as analytical patterns rather than causal proof.

While qualitative coding is described, inter-coder reliability or independent validation procedures are not reported, raising concerns regarding analytical subjectivity.

- We agree with your comment and have added clarification in Section 3.2, page 12.

Several sections rely heavily on narrative description of incidents, with limited analytical abstraction beyond mapping events to SCCSS categories.

- Thank you for this comment. While the case stories provide essential grounding, the paper goes well beyond simple storytelling by theorizing how supply chain failures actually work. We introduced the concept of Synergy Dependency to show that external information-sharing is only useful if a company's internal teams are ready to act on that data. We also re-imagined Points of Penetration (PoPs) as the active sites of "cyber-physical conversion," explaining exactly how a digital hack turns into a real-world operational halt. Finally, by identifying four specific propagation mechanisms, we have provided a clear framework for understanding how risk spreads through different industries. This moves the SCCSS model from a static checklist to a dynamic tool for diagnosing network resilience.

Quantitative indicators (e.g., recovery time, financial loss, operational downtime) are inconsistently reported across cases, making cross-case comparison less systematic.

- We acknowledge that the quantitative indicators like exact financial losses or recovery hours vary between cases, but that is a known hurdle when working with public data—companies often keep those specific details under wraps to manage legal liability and their reputation. Because of this inherent visibility bias in public reporting, we intentionally designed this as a qualitative multi-case study focused on analytical generalization rather than a statistical one. Instead of relying on inconsistent dollar amounts to prove a point, we utilized replication logic to see if the same governance failures consistently led to the same types of systemic shutdowns across different sectors. By focusing on the propagation mechanisms—the actual "machinery" of the failure—we can still draw conclusions about resilience even when the exact balance sheets for every incident are not publicly available.

- Moreover, we have added Table 5 (Quantitative Indicators of Supply Chain Cyberattacks) in Section 5.8 to summarize some of the quantitative indicators we manage to obtain.

The discussion emphasizes well-known cybersecurity practices (Zero Trust, MFA, vendor audits),

but offers limited actionable differentiation from existing cybersecurity guidelines (e.g., NIST SP 800-161).

- While NIST SP 800-161 provides a solid technical baseline, our paper focuses on the internal coordination agility that determines if those tools actually work in crisis. We introduce Synergy Dependency, proving that even the best threat intelligence is effectively useless if your internal team lacks the authority to act on it immediately. We provide four specific propagation mechanisms—like Network Flattening and Operational Coupling—that can be plugged into digital twin simulations to stress-test real-world production lines.

The manuscript would benefit from a clearer visual synthesis (e.g., conceptual diagram or summary table) illustrating cross-case patterns of vulnerability and resilience.

- We thank you for the insight regarding the need for a clearer visual synthesis of our findings. In response, we have added Table 4 to consolidate the cross-case patterns of vulnerability and resilience into a single, comprehensive reference. This table provides a clear visual link between the initial entry vectors, the escalation mechanisms, and the specific governance gaps that allowed these breaches to spread. We believe this expanded synthesis directly addresses the reviewer's request for a visual summary that illustrates systemic patterns across diverse industries.

Some references rely on technical blogs or news outlets rather than peer-reviewed literature, which weakens academic rigor.

- While we agree that technical blogs or news coverage might not have the same rigor as peer-reviewed, they are necessary to obtain detailed and timely coverage of cyberattack incidents. We try to use peer-reviewed papers as information sources whenever possible. For example, in the revised manuscript, we have substituted a blog article with Plachkinova and Maurer (2018) published in Journal of Information Systems Education and Pigni et al. (2018) published in Journal of Information Technology Teaching Cases.

The conclusion largely reiterates earlier findings without sufficiently articulating broader theoretical implications for supply chain governance research.

- We agree with your comment and formally incorporated Supply Chain Governance theory in the revised manuscript, which strengthens the conclusion.

Language in several sections is managerial or descriptive rather than analytical, and could be tightened to improve academic tone.

- Thank you for the suggestion. We have made efforts to streamline the language for academic audience.

Response to Reviewer #2 Comments

We thank you and the review team for the detailed reviews that have helped us greatly in revising the manuscript. Following your suggestions, we have made significant changes to our manuscript. Together with the new version of the manuscript, we are also submitting revision notes for the review team. In our revision, we have made conscientious efforts to address your concerns. We believe that by addressing the review team's concerns, we now have a much stronger manuscript. In the following paragraphs, the comments that you raise are reproduced verbatim in *italics*. Our responses to the comments are in regular font. We have also submitted a revised manuscript with tracked changes.

Summary of the research and overall impression: This manuscript does a good job of examining supply chain cyberattacks using a qualitative multi-case study approach. The authors analyze seven real-world cyberattack incidents across multiple industries and apply the Supply Chain Cyber Security System (SCCSS) framework to identify common attack vectors, propagation mechanisms, and governance failures. The study aims to understand how weaknesses in IT systems, organizational practices, and supply chain governance contribute to cyberattack entry and spread.

- Thank you for the nice summary of our manuscript.

The manuscript addresses a relevant and timely research problem and uses an appropriate qualitative methodology to achieve this. It is kind of a tedious read, but a good one ultimately.
Overall, the study is technically sound and contributes useful insights into supply chain cybersecurity governance. However, several minor issues should be addressed to improve methodological clarity and ensure full compliance with journal formatting standards, and these will be addressed subsequently.

- Thank you for the relevance and timeliness of our manuscript. We have revised the manuscript according to your helpful comments and complied with journal formatting standards.

Major Issues
1. Reference numbering and formatting should be corrected
Unless I am missing something, and please let me know if I am, but References should be numbered strictly in order of first appearance. For example, early in the Introduction, references are cited out of sequence, which suggests that numbering was not properly synchronized. These Numbering inconsistencies should be addressed in full compliance with PLOS ONE reference style (Vancouver).

- Thank you for pointing out this issue. We have re-numbered the references as they are cited in order at first mention. We have also formatted the references according to PLOS ONE reference style (Vancouver).

2. Clarification of coding reliability and consistency
The manuscript describes the coding process used to enhance reliability, which strengthens the analysis. However, the manuscript does not clearly explain whether multiple researchers independently coded the data or how coding disagreements were resolved. The authors should

provide additional clarification on how coding consistency and reliability were ensured. This is the chief reason for the "partly" answer in question 1.

- We thank the reviewer for highlighting this important methodological detail. We entirely agree that explicitly detailing the independent validation and reconciliation procedures strengthens the transparency and credibility of our qualitative analysis. We revised Section 3.2 to state that two researchers independently coded the cases and resolved disagreements through discussion using the raw sources and SCCSS definitions.

minor issues
1. Relying fully on secondary sources (such as reports and news articles) can be tricky. While this is acceptable for case studies, it limits the strength of the conclusions.

- We appreciate your evaluation of our data sources. In our original manuscript, we acknowledged this limitation in Section 6.3 (Limitations and Future Research Directions). To clarify this limitation, the revised manuscript now explicitly details the specific limitations associated with our data types, acknowledging that journalistic accounts may harbor interpretive bias, forensic analyses often narrow their focus to technical flaws at the expense of governance context, and corporate disclosures are inherently designed to protect reputational assets and may omit sensitive operational details. We believe this comprehensive reflection provides transparent and rigorous boundaries for our findings.

2. The manuscript is generally well written and easy to understand, but there are minor grammatical issues and occasional repetitive phrasing. These can be corrected during revision.

- We proofread the revised manuscript and reduced repetitive or informal phrasing.

Conclusion: This is a technically sound paper, and analysis has been done well for the most part. It, however requires revisions before it is quite ready for publication.

- Thank you for your favorable recommendation. Your helpful comments are greatly appreciated, and we have revised the manuscript accordingly.

Response to Reviewer #3 Comments

We thank you and the review team for the detailed reviews that have helped us greatly in revising the manuscript. Following your suggestions, we have made significant changes to our manuscript. Together with the new version of the manuscript, we are also submitting revision notes for the review team. In our revision, we have made conscientious efforts to address your concerns. We believe that by addressing the review team's concerns, we now have a much stronger manuscript. In the following paragraphs, the comments that you raise are reproduced verbatim in *italics*. Our responses to the comments are in regular font. We have also submitted a revised manuscript with tracked changes.

In the abstract and conclusion, the contribution of this paper is not well presented. In the conclusion and abstract, highlight the novelty of the paper.

- Per your comment, we have revised the abstract and conclusion to highlight the contribution and novelty of the paper.

The introduction is weak and should include the research question, the aim of the paper and the contribution.

- Per your comment, we have moved Section 3.1 (research questions) to the end of Section 1.

In related work…. Many researchers work on this idea. What is really the novelty as compared to other studies? What is the new and the difference between the previous works and present work?

- Our novelty lies in framing supply chain cyberattacks as a system-level propagation process rather than isolated technical events. While previous studies often focus on physical reliability or mere technical analyses, we utilize the SCCSS framework to map how risks transition from digital entry points to physical operational paralysis. The primary difference from existing literature is the empirical identification of Governance Synergy. We demonstrate that relational mechanisms, such as information sharing, remain "dormant" assets unless activated by internal organizational coordination. Furthermore, this study extends the SCCSS model by defining PoPs (technology, human resources, and physical processes) as the mechanisms through which cyber incidents escalate across a network. By categorizing distinct propagation drivers such as Network Flattening and Operational Coupling, our study provides a nuanced, governance-based roadmap for resilience that traditional, compliance-oriented models lack.

Improve the quality of literature along with the latest literature. The explanation of the related work needs to be criticized and improved in general. What about last updating in this topic and new references from 2019-2025? The survey of existing literature is not sufficient.

- Thank you for the suggestion. We strengthened the literature review with recent studies from 2019–2025 and integrated them into the related-work discussion:

    1. Kumar S, Mallipeddi RR. Impact of cybersecurity on operations and supply chain management: Emerging trends and future research directions. Production and Operations

Management. 2022;31(12):4488–500.

2. Jeong S, Rogers Z, Choi TY. Strange dance partners: supply chain cyberattacks and chained vulnerability. Journal of Operations Management. 2025;71(6):763–85.

3. Pérez-Morón J. Eleven years of cyberattacks on Chinese supply chains in an era of cyber warfare, a review and future research agenda. Journal of Asia Business Studies. 2022;16(2):371–95.

- Moreover, we want to bring to your attention that the previous version of the manuscript cites the following references from 2019-2025, which we keep in the revised manuscript:

1. Reuben-Owoh B, Haig E. A Systematic review of Voluntary cybersecurity standards and Frameworks. International Journal of Information Security. 2025;24(5).

2. Durugbo CM, Al-Balushi Z. Supply chain management in times of crisis: a multi-case study', Production Planning & Control, 2024;36(11):1529-1557.

3. Ilori O, Nwosu N. Third-Party Vendor Risks in IT Security: A Comprehensive Audit Review and Mitigation Strategies, World Journal of Advanced Research and Reviews, 2024;22(3):213-224.

4. Konecka S, Bentyn Z. Cyberattacks as Threats in Supply Chains, European Research Studies Journal, 2024;27(3):778-796.

5. Looi JCL, Looi RCH, Maguire PA, Kisely S, Bastiampillai T, Allison S. Psychiatric electronic health records in the era of data breaches - What are the ramifications for patients, psychiatrists and healthcare systems?, Australasian Psychiatry, 2024;32(2):121-124.

6. Lu Q, Jiang Y, Wang Y. The effects of supply chain governance on supply chain resilience based on information processing theory, Industrial Management & Data Systems, 2024;124(1):291–318.

7. Mittal M. Colonial Pipeline Cyberattack Drives Urgent Reforms in Cybersecurity and Critical Infrastructure Resilience, International Journal of Oil, Gas and Coal Engineering, 2024;12(5):106-119.

8. Xu L, Li Y, Lin Y, Tang C, Yao Q. Supply chain cybersecurity investments with interdependent risks under different information exchange modes. International Journal of Production Research. 2023;62(6):2034–59.

9. Li B, Liu S. When to swing into high gear? A time-limit approach to problem escalation. IISE Transactions. 2023;55(6): 644–655.

10. Li B, Tan Z, Arreola-Risa A, Huang Y. On the Improvement of Uncertain Cloud Service Capacity, International Journal of Production Economics, 2023:258(1):108779.

11. Bonatto F, Resende LMMD, & Pontes J. Supply chain governance: a conceptual model, Journal of Business & Industrial Marketing, 2022;37(2), 309-325.

12. Crosignani M, Macchiavelli M, Silva AF. Pirates without borders: The propagation of cyberattacks through firms' supply chains. Journal of Financial Economics. 2022;147(2):432–48.

13. Li B, Arreola-Risa A. On minimizing downside risk in make-to-stock, risk-averse firms Naval Research Logistics (NRL). 2021;68(2):199–213.

14. Martinez J, Duran JM. Software Supply Chain Attacks, a Threat to Global Cybersecurity: SolarWinds' Case Study, International Journal of Safety and Security Engineering, 2021;11(5):537-545.

15. Ghadge A, Weiß M, Caldwell ND, & Wilding, R. Managing cyber risk in supply chains: a review and research agenda, Supply Chain Management: An International Journal, 2020;25(2):223-240.

16. Rybeck T. Cybersecurity lessons from the field. Defense Transportation Journal, 2020;76(4):22-23.

It would be useful to include in the Introduction of the paper some discussion on other possible real applications of the obtained results.

- According to your suggestion, we have added a paragraph at the end of Section 1. We have also expanded the managerial implications in Section 6.2 to discuss some real applications.

Weak conclusion. Conclusion should be more specific with improvement writing quality.

- Per your comment, we have improved the conclusions sections with writing enhancements.

A suggestion for future work should be added in the conclusion section.

- In the original manuscript, we included a paragraph on future research directions in Section 6.3 (Limitations and Future Research Directions). Per your comment, we have expanded suggestions for future work in the revised manuscript.

Rewrite the references according to journal template. Please strictly follow the instructions to the format specified in the journal template for preparing the paper

- In the revised manuscript, we have strictly follow the instructions to the format specified in the journal template.

The format and English writing of this paper should be improved. The paper needs language revision.

- In the revised manuscript, we have performed language revision to improve the English writing of this paper.

Response to Reviewer #4 Comments

We thank you and the review team for the detailed reviews that have helped us greatly in revising the manuscript. Following your suggestions, we have made significant changes to our manuscript. Together with the new version of the manuscript, we are also submitting revision notes for the review team. In our revision, we have made conscientious efforts to address your concerns. We believe that by addressing the review team's concerns, we now have a much stronger manuscript. In the following paragraphs, the comments that you raise are reproduced verbatim in *italics*. Our responses to the comments are in regular font. We have also submitted a revised manuscript with tracked changes.

This study examines Supply Chain Cyberattacks (SCCAs) through a qualitative multi-case analysis of seven major cyber incidents across industries such as retail, logistics, energy, and healthcare. Using the Supply Chain Cyber Security System (SCCSS) framework, the authors analyze how vulnerabilities in IT systems, organizational governance, and supply chain relationships contribute to cyberattack entry points and propagation. The findings show that most attacks originate from trusted third-party connections, and their operational impact is amplified by weak internal controls and poor coordination across systems. The study concludes that improving cyber resilience requires cross-organizational governance mechanisms, including stronger third-party risk management, network segmentation, and proactive information sharing among supply chain partners.

- Thank you for the nice summary of our manuscript.

The introduction concludes by stating that the study investigates the propagation of supply chain cyberattacks (SCCAs). However, the research questions span several distinct phenomena, including attack entry points (RQ1), inter-system escalation (RQ2), and recovery through intelligence sharing (RQ3). Only RQ2 directly addresses propagation. The manuscript would benefit from clarifying the conceptual relationship among these questions and explaining how they collectively contribute to understanding cyberattack propagation within the SCCSS framework.

- We thank the reviewer for this insightful critique. We agree that the term "propagation" required a more robust conceptual definition to demonstrate how the three research questions (RQs) function as an integrated analytical whole. In the revised manuscript, we revised the Introduction to define propagation as a lifecycle consisting of inception, transmission, and interruption, which clarifies the role of all three research questions. Specifically, we have implemented the following changes to clarify the relationship among the RQs:

  - ✧ Introduction of the Propagation Lifecycle: We have added a transitional section at the end of the Introduction to frame propagation as a dynamic process consisting of inception, transmission, and interruption. This allows us to explain that while lateral spread (RQ2) is a core component, the full trajectory of propagation cannot be understood without identifying its origin (RQ1) and its eventual termination (RQ3).

  - ✧ Theoretical Refinement of Research Questions: To strengthen the conceptual link, the RQs have been updated to incorporate Supply Chain Governance (SCG) theory. This

move ensures that each stage of the propagation lifecycle is tied to a specific governance mechanism:

RQ1 (Inception phase): Investigates how failures in contractual governance facilitate the initial entry of attacks.

RQ2 (Transmission phase): Examines how a deficit in internal governance exacerbates the lateral spread and operational magnitude of the breach.

RQ3 (Interruption phase): Explores how relational governance and proactive intelligence-sharing influence the speed of containment and systemic recovery.

- Structural Realignment: Per the suggestion to improve clarity, the research questions and their conceptual justification have been moved to the end of Section 1 (Introduction). This establishes the "propagation lifecycle" as the central roadmap for the paper, directly linking the industrial problem to our theoretical inquiry.

By framing the RQs in this manner, we demonstrate that all three are essential for a holistic understanding of how cyberattacks move through and are eventually expelled from the SCCSS.

Throughout the manuscript, the study is framed as investigating the propagation of supply chain cyberattacks (SCCAs). However, the conclusion does not clearly articulate what specific insights the study offers regarding propagation mechanisms beyond the observation that attacks often originate from third-party entry points and spread through poorly coordinated systems. The authors could clarify the concept of propagation by specifying the mechanisms through which cyber incidents escalate across systems. Explicitly summarizing these mechanisms in the conclusion would help consolidate the study's contribution and provide a clearer conceptual understanding of how SCCAs spread across supply chain networks.

- We appreciate the reviewer's suggestion to explicitly summarize the propagation mechanisms identified in our research. We agree that a clearer conceptualization of how incidents escalate is vital for consolidating the study's contribution. In the revised manuscript, we revised the Conclusion to summarize four propagation mechanisms: Network Flattening, Alert Paralysis, Operational Coupling, and Relational Weaponization as outlined in Section 6.1. These mechanisms provide a structured framework for understanding how SCCAs transition from initial entry to systemic disruption.

The paper introduces Points of Integration (technology, human resources, and physical processes) as mechanisms connecting the three SCCSS subsystems. However, the relationship between these PoPs and the subsystem architecture in Table 1 is not clearly articulated. It remains unclear how the PoPs operationalize or interact with the IT, organizational, and supply chain security subsystems. Please consider providing an explicit mapping or conceptual diagram that would substantially improve the clarity of the framework.

- Per your comment, we have added a paragraph in Section 4 to improve the clarity of the framework. We have also merged Section 2.4 into Section 4 to avoid stating the research

framework in different sections.

It is unclear whether the selected cases represent typical supply chain cyberattacks or simply the most visible ones. Without systematic selection criteria, the findings may reflect selection bias rather than generalizable patterns. The authors could strengthen methodological transparency by explicitly describing the case selection criteria and explaining why these seven cases provide appropriate analytical coverage of supply chain cyber risks. For example, the authors might clarify whether cases were selected to represent variation in industries, attack vectors, governance failures, or supply chain structures. Providing a short table summarizing selection rationale or linking case characteristics to the research questions would improve confidence in the robustness of the case design.

- Your comment is appreciated. We revised Section 3.2 to describe the candidate-case pool on page 10, the purposive sampling criteria, and the rationale for selecting the final seven cases.

The manuscript states that deductive thematic coding was conducted using the SCCSS framework to classify empirical observations into IT, organizational, and supply chain subsystems. However, the description of the coding process remains relatively brief and does not fully clarify how the analysis was conducted in practice. It is unclear whether multiple researchers participated in the coding process, how coding consistency was ensured, or whether any form of intercoder agreement assessment was performed. Without additional detail, it is difficult for readers to assess the reliability and replicability of the qualitative analysis. The authors could enhance methodological rigor by providing more detail on the coding procedure. For instance, clarifying how many researchers participated in coding, whether the coding scheme was iteratively refined, and how disagreements were resolved would strengthen transparency. If applicable, reporting an intercoder agreement measure or describing a consensus-based coding process would further reinforce the credibility of the analytical procedure. Even a brief appendix illustrating sample coded excerpts could significantly improve reproducibility.

- Your comment is appreciated. We expanded Section 3.2 (pages 11 to 12) to describe the three-step coding procedure, the independent coding process, and the consensus-based resolution of disagreements.

Several core concepts: inter-system coordination, attack propagation, governance robustness, and recovery speed, play a central role in the cross-case analysis. However, these constructs are primarily discussed in descriptive terms, and their operationalization across cases is not always clearly specified. As a result, it becomes difficult to determine how these concepts were consistently assessed across the seven incidents or how the authors compared cases. To improve analytical clarity, the authors could more explicitly define how key constructs were operationalized in the case analysis. For example, indicators such as attack entry vector, internal propagation mechanism, detection delay, or recovery duration could be consistently mapped across cases. A summary table that operationalizes these constructs and shows how they were observed in each case would help readers understand how the cross-case comparisons were conducted and would strengthen the overall methodological transparency of the study.

- We appreciate this feedback and agree that showing the "analytical machinery" behind our case

studies helps move the paper beyond simple storytelling. To make our process more transparent, we have added a new Cross-Case Operationalization Table (Table 4) that explicitly maps how we measured core concepts like Attack Entry Vectors, Detection Delay, and Recovery Duration across all seven incidents. By consistently defining these indicators—such as tracking "Detection Delay" as the time from initial infiltration to discovery—we can clearly compare the 15-month dwell time at SolarWinds against the near-instant operational paralysis at Maersk. This structured approach proves that our conclusions about "Inter-system Coordination" and "Governance Robustness" aren't just descriptive, but are based on specific, observable failures in how IT alerts and organizational policies interacted during a crisis. Every quantitative indicator used in this comparison is pulled directly from the forensic reports and investigative data already cited in the manuscript, ensuring that the entire analysis is grounded in verifiable evidence.

The manuscript claims that it "empirically validates the Supply Chain Cyber Security System (SCCSS) framework" and makes a "distinct theoretical contribution" by demonstrating that cyber risk is a systemic property emerging from the interdependence of IT, organizational, and supply chain subsystems. However, the empirical analysis primarily illustrates how well-known cyber incidents can be interpreted through the SCCSS lens, rather than demonstrating clear theoretical advancement. The cases show that vulnerabilities may arise from IT weaknesses, organizational failures, and supply chain relationships, those insights are broadly consistent with existing cybersecurity and supply chain risk management literature. The study appears to apply an existing framework to multiple cases, but it is less clear how the findings extend, refine, or challenge the framework in a theoretically meaningful way. The authors should strengthen the theoretical contribution by explicitly articulating how the case evidence refines the framework, not simply confirming it.

- We appreciate the reviewer's point and revised Section 6.1 to clarify how the study refines SCCSS in three ways: governance synergy, transmission-site interpretation, and propagation mechanisms.

  1. The Governance Synergy Requirement (Refinement): The original SCCSS framework posits that subsystems are interconnected. However, our analysis of the SolarWinds and MediBank cases reveals a specific conditional dependency: Relational Governance (Supply Chain Subsystem) is fundamentally insufficient for resilience without the Internal Governance (Organizational Subsystem) to act upon it. We refine the framework by theorizing that Internal Governance serves as the "operational switch" that determines whether external intelligence translates into systemic defense.

  2. Operationalization of Points of Integration (PoPs) (Extension): While the SCCSS model identifies PoPs (Technology, Human, Physical) as connection points, it does not define their functional role in propagation. This study extends the framework by defining PoPs as the specific "transmission sites" for cyber-physical disruption. For example, the Toyota case demonstrates how the Technology PoP (the Kanban system) acts as a mechanism that converts a digital failure into total physical paralysis.

  3. Challenging the "Efficiency-Resilience" Paradox (Challenge): We challenge the

traditional focus of Supply Chain Risk Management (SCRM) on physical reliability by proving that physical "Lean" optimizations (Supply Chain subsystem) create rigid digital dependencies that accelerate the velocity of cyber-propagation.

These revisions move the study beyond mere interpretation and offer a more nuanced, proposition-driven refinement of the SCCSS framework.

The manuscript addresses an important and timely topic and provides insightful case analyses of major supply chain cyber incidents. However, several aspects of the methodological design and theoretical positioning require further clarification. In particular, the case selection rationale, transparency of the coding procedure, and operationalization of key constructs should be strengthened.

- Per your comment, we have elaborated on the case selection process, the coding procedure, and the operationalization of key constructs.

Additionally, the manuscript would benefit from a more cautious articulation of its theoretical contribution. I wish the authors the best in revising the manuscript and look forward to seeing how the study develops in the next iteration.

- Thank you for your time and consideration. We hope you find the revised manuscript acceptable.
