# Supplementary material for: Cyberattacks in supply chains: A multi-case study
Source: PLoS One. 2026 May 22;21(5):e0350010. doi: 10.1371/journal.pone.0350010 (PMC13196919; doi:10.1371/journal.pone.0350010)
Supplement: Attachment — Submitted filename: Response to R2 reviews.docx [file pone.0350010.s003.docx]

**Response to Editor Comments**

We thank you and the four referees for the detailed reviews that have helped us greatly in revising the manuscript. Following your suggestions, we have made significant changes to our manuscript. Together with the new version of the manuscript, we are also submitting revision notes for the review team. In our revision, we have made conscientious efforts to address your concerns. We believe that by addressing the review team’s concerns, we now have a much stronger manuscript. In the following paragraphs, the comments that you raise are reproduced verbatim in *italics*. Our point-by-point responses to the editorial comments are in regular font. We have also submitted a revised manuscript with tracked changes.

1. *While the reviewers have expressed generally positive views regarding the relevance of the topic and the clarity of the manuscript, I find that the paper, in its current form, requires a few more revisions before it can be considered for publication. I am therefore inviting you to submit a major revision.*

- We thank you for handling the manuscript with care. We have revised the manuscript extensively to address your comments.

2. *Your study addresses an important and timely issue (cyber risk propagation in supply chains) and presents a well-structured qualitative multi-case analysis of seven high-profile incidents. The manuscript is clearly written, logically organised, and grounded in relevant literature. The use of the SCCSS framework to structure the analysis is appropriate, and the comparative perspective across cases has the potential to offer valuable insights.*

- Thank you for the summary.

3. *However, several concerns must be addressed to ensure that the manuscript meets the standards of methodological rigor and interpretative balance required for publication.*

- We have revised the manuscript extensively to address your concerns on methodological rigor and interpretative balance.

4. *The manuscript presents strong theoretical claims, however, these contributions are not sufficiently substantiated by the analysis. The current evidence supports the identification of recurring patterns across cases but does not justify the level of theoretical advancement claimed. I encourage you to recalibrate the framing of your contributions, positioning them more cautiously as empirically grounded insights or analytical interpretations rather than formal theoretical extensions...or to stress the discussion and the result analysis in order to allign them with your theoretical claims.*

- We appreciate your valuable comment. Per your comment, we have recalibrated the framing of contributions and made the following changes:
  - In the abstract, we have changed the contribution of the paper from “refines and extends the SCCSS framework” to “offers empirically grounded insights that adapt the SCCSS framework”. We have also changed “The findings advance supply chain governance theory” to “The findings contribute analytical interpretations to supply chain governance theory”.
  - In the introduction section on page 4, we have changed “advances supply chain governance theory by moving beyond a static, component-based view” to “This study offers analytical interpretations that inform supply chain governance theory by moving beyond a static, component-based view”.
  - In Section 6.1 on page 39, we have changed “This study makes a distinct theoretical contribution by moving beyond the interpretation of cyber incidents to a structural refinement of the SCCSS framework.” to “This study offers empirically grounded analytical interpretations that build upon the SCCSS framework.” On page 39 and 41, the word “dictate” is changed to “indicate”.

5. *Section 5.9 (“Proposition Verification”) reads primarily as a confirmatory narrative in which all propositions are reported as “aligned.” This approach does not provide a sufficiently critical or analytical evaluation. Please revise this section to engage more critically with the evidence, acknowledge ambiguity, boundary conditions, or partial support, avoid presenting propositions as universally confirmed.*

- We appreciate your insightful observation. We agree that presenting the theoretical propositions as universally "aligned" overly simplified the complex realities of supply chain cyber risk and detracted from the analytical depth of the study. To provide a more critical evaluation, we have thoroughly rewritten Section 5.9 to remove the confirmatory framing. Instead of simply validating each proposition, the revised section now interrogates the empirical evidence to identify boundary conditions, instances of partial support, and the practical limitations of specific governance mechanisms. For example, the text now critically examines how contractual governance fundamentally fails against sophisticated embedded software threats, and how external information sharing can create a false sense of security if not matched by internal operational readiness. By explicitly acknowledging these ambiguities, we believe the section now offers a much more rigorous and nuanced theoretical discussion.

6. *While the manuscript includes a cross-case synthesis, the comparative analysis remains largely descriptive. I encourage you to deepen this section by more explicitly contrasting cases, highlighting both differences and similarities, and clarifying under what conditions particular mechanisms or outcomes emerge.*

- Thank you for the helpful feedback. We agree that directly comparing the cases makes the paper much stronger. To address this, we revised Section 5.9 on pages 38-39 to explicitly contrast the seven incidents across three main dimensions.
  - First, we looked at the size of the collapse. We contrasted Maersk's total global shutdown with the smaller, contained disruptions at Target and Colonial Pipeline. This shows that a flat network only causes a complete failure if an organization's internal controls are weak.
  - Second, we examined how fast the attacks spread. By comparing NotPetya's instant crash to the 15-month SolarWinds breach, we highlight that quick information sharing and strong internal monitoring are required to slow down a threat.
  - Finally, we compared physical resilience. We contrasted Toyota’s rigid Just-in-Time system—where one supplier's issue stopped everything—with Maersk’s ability to switch to manual paper records. This proves that digital safety depends heavily on how flexible the physical supply chain is. We believe these direct comparisons clearly show the exact conditions that cause attacks to either spread or be stopped.

7. *Although the manuscript acknowledges the use of secondary data and potential biases, these limitations should be more explicitly integrated into the interpretation of findings.*

- Thank you for pointing out this issue. As you have correctly pointed out, we have already discussed the limitations of using secondary data
  - On page 11: “Given the reliance on secondary data, we employed data triangulation to validate the accuracy of the events. Technical details regarding attack vectors were sourced specifically from forensic reports (e.g., FireEye, Microsoft) and government filings (e.g., CISA, Senate investigations), while organizational impacts and timelines were cross-referenced with corporate press releases and investigative journalism. This multi-source approach mitigated the potential bias of any single report and ensured that the technical reality of each incident was accurate before analysis began.”
  - On pages 43-44: “First, while this study employs data triangulation to synthesize event timelines, relying entirely on publicly available secondary sources introduces inherent methodological constraints. Each source type carries specific biases: investigative journalism may contain interpretive bias, technical reports often overemphasize technical vulnerabilities at the expense of governance context, and corporate disclosures frequently omit sensitive operational details to manage public relations and legal liability. Although cross-verifying these disparate sources helps construct a more objective narrative, it cannot completely eliminate the blind spots inherent in relying exclusively on external, post-hoc reporting rather than direct organizational access.”
- To address your concern, we have revised the interpretation of findings by adding a paragraph on pages 34-35: “Because this study draws on secondary data from publicly documented incidents, the findings are shaped by reporting and visibility biases. Accordingly, the identified propagation mechanisms and governance patterns should be read as robust analytical patterns across the selected cases, but not as fully representative of all supply chain cyberattacks.”

8. *Some of the managerial and policy implications (e.g., applications to insurance models, digital twin simulations, and regulatory frameworks) extend beyond what is directly supported by the empirical analysis. Please ensure that all implications are clearly grounded in the presented evidence, and consider moderating or removing those that are more speculative in nature.*

- We have removed the following arguments in the revised manuscript:
  - Page 5 and page 42 for insurance models
  - Page 5 and page 43 for digital twin simulations
- We have strengthened the following argument in the revised manuscript:
  - Page 5 and page 43 for regulatory frameworks, by mentioning the U.S. Cybersecurity Information Sharing Act of 2015.

9. *In summary, the manuscript has clear potential but requires revisions to ensure that its claims are appropriately supported and that the analysis meets the expected level of rigour. I would be happy to consider a revised version that addresses the points outlined above.*

- We thank you for handling the manuscript with care. We have revised the manuscript extensively to address your comments, and these revisions have strengthened the methodological transparency and nuanced interpretation of governance mechanisms.

10. *In order to help you in your last effort and to better align the manuscript to the journal, I list some references useful for your work:*
    - *10.1111/itor.70072*
    - *10.1371/journal.pone.0335128*
    - *10.1016/j.jik.2026.100939*
    - *10.1371/journal.pone.0344098*
    - *10.1109/TEM.2025.3648054*
    - *10.3390/systems14020132*

- We sincerely appreciate your guidance in providing these highly relevant references. We have carefully reviewed each suggested article and integrated them into the manuscript to better align our work with the current academic discourse on supply chain cybersecurity. Specifically, the recent articles examining software supply chains and causal relationships have been incorporated into our literature review to strengthen the discussion there. The insights regarding cybersecurity outsourcing and dynamic risk assessment frameworks from these suggested publications provided excellent context for our analysis of third-party risk management and contractual governance. These additions have profoundly enriched our theoretical positioning, and we are extremely grateful for the targeted recommendations.
  - On page 5, we have added “The inherent multi-level structures of software supply chains introduce novel challenges in network security, where risk contagion mechanisms and complex causal relationships dictate the speed and severity of vulnerability propagation” for Cai et al. (2025) and Adem et al. (2026).
  - On page 6, we have added “Because modern supply chains function as complex adaptive systems, cyber vulnerabilities cannot be evaluated in isolated nodes; rather, dynamic risk assessment frameworks are required to map the cascading interdependencies and mitigate the systemic uncertainties inherent in digitalized outsourcing” for Dey et al. (2025).
  - On page 7, we have added “Moreover, the effectiveness of cybersecurity outsourcing is heavily dependent on strategic organizational alignment.” For Annarelli et al. (2026) and Plevnik et al. (2026).
  - On page 14, we have added “This is because robust technology architectures must be inherently adaptive, meaning that static security perimeters are fundamentally insufficient without dynamic technological coordination across inter-organizational boundaries” for Luo et al. (2025).
  - We have updated the numbering of references in the revised manuscript accordingly.

**References:**

Cai H, Xiong Q, Lian S. Research on network security vulnerability risk contagion in software supply chain based on system dynamics. PLoS ONE. 2025;20(11):e0335128.

Adem A, Çakıt E, Dağdeviren M, Mrugalska B, Karwowski W. Understanding security challenges in the software supply chain through causal relationships. PLoS ONE. 2026;21(3):e0344098.

Annarelli A, Colabianchi S, Nonino F, Oropallo E, Palombi G. Is cybersecurity outsourcing always effective? Strategic organisational insights and knowledge in the era of digitalisation. Journal of Innovation & Knowledge. 2026;14:100939.

Plevnik M, Rupnik B. Developing DEFCHAIN: a dynamic framework for cybersecurity risk assessment in military supply chains. Systems. 2026;14(2):132.

Dey SK, Kundu K, Das P. Digital and greening strategies for financial resilience in a three‐echelon supply chain. International Transactions in Operational Research. 2025;33(4):2289–2324.

Luo S, Jiang X, Feng L, Fu X. E-Tailers facing Cyberattacks: Impacts of risk attitudes. IEEE Transactions on Engineering Management. 2026;73:949–961.
